# Renal dysfunction in STEMI-patients undergoing primary angioplasty: higher prevalence but equal prognostic impact in female patients; an observational cohort study from the Belgian STEMI registry

**DOI:** 10.1186/1471-2369-14-62

**Published:** 2013-03-18

**Authors:** Sofie A Gevaert, Dirk De Bacquer, Patrick Evrard, Marc Renard, Christophe Beauloye, Patrick Coussement, Herbert De Raedt, Peter R Sinnaeve, Marc J Claeys

**Affiliations:** 1 Department of Cardiology, Ghent University Hospital, Ghent, Belgium; 2 Department of Public Health, Ghent University, Ghent, Belgium; 3 Department of Intensive Care, Université Catholique de Louvain, Yvoir, Belgium; 4 Department of Cardiology, Erasme Academic Hospital, Brussels, Belgium; 5 Université Catholique de Louvain, Department of Cardiology, Brussels, Belgium; 6 Department of Cardiology, Hospital St. Jan, Bruges, Belgium; 7 Cardiovascular centre, OLV Hospital Aalst, Aalst, Belgium; 8 Department of Cardiovascular diseases, University Hospitals Leuven, Leuven, Belgium; 9 Department of Cardiology, University Hospital Antwerp, Edegem, Belgium

**Keywords:** ST-segment elevation myocardial infarction (STEMI), Estimated glomerular filtration rate (eGFR), CKD-EPI, Renal dysfunction, Gender, In-hospital mortality, Primary angioplasty

## Abstract

**Background:**

Mortality in female patients with ST-segment elevation myocardial infarction (STEMI) undergoing primary angioplasty (pPCI) is higher than in men. We examined gender differences in the prevalence and prognostic performance of renal dysfunction at admission in this setting.

**Methods:**

A multicenter retrospective sub-analysis of the Belgian STEMI-registry identified 1,638 patients (20.6% women, 79.4% men) treated with pPCI in 8 tertiary care hospitals (January 2007-February 2011). The estimated glomerular filtration rate (eGFR) was calculated using the CKD-EPI equation. Main outcome measure was in-hospital mortality.

**Results:**

More women than men suffered from renal dysfunction at admission (42.3% vs. 25.3%, p < 0.001). Mortality in women was doubled as compared to men (9.5 vs. 4.7%, OR (95% CI) = 2.12 (1.36-3.32), p<0.001). In-hospital mortality for men and women with vs. without renal dysfunction was much higher (10.7 and 15.3 vs. 2.3 and 2.4%, p < 0.001). In a multivariable regression analysis, adjusting for age, gender, peripheral artery disease (PAD), coronary artery disease (CAD), hypertension, diabetes and low body weight (<67 kg), female gender was associated with renal dysfunction at admission (OR (95% CI) 1.65 (1.20-2.25), p = 0.002). In a multivariable model including TIMI risk score and renal dysfunction, renal dysfunction was an independent predictor of in-hospital mortality in both men (OR (95% CI) = 2.39 (1.27-4.51), p = 0.007) and women (OR (95% CI) = 4.03 (1.26-12.92), p = 0.02), with a comparable impact for men and women (p for interaction = 0.69).

**Conclusions:**

Female gender was independently associated with renal dysfunction at admission in pPCI treated patients. Renal dysfunction was equally associated with higher in-hospital mortality in both men and women.

## Background

It has been demonstrated that women with STEMI undergoing primary PCI (pPCI) have higher odds for in-hospital mortality than men. Some authors demonstrated that this difference is likely explained by their older age and baseline comorbidities (especially hypertension and diabetes) [1-5], while other authors demonstrated a sustained mortality difference even after adjustment for appropriate confounders [6-8]. Chronic kidney disease, even mild, is associated with increased cardiovascular mortality [9]. More recently it was demonstrated that renal dysfunction is independently associated with in-hospital mortality in STEMI patients treated with pPCI [10-12].

Data on gender differences in prevalence of renal dysfunction at admission in pPCI treated STEMI patients are scarce and have seldom been accounted for when evaluating gender differences in outcome, furthermore different definitions (serum creatinine levels vs. estimated Glomerular Filtration Rate (eGFR) values) and different methods of estimating creatinine clearance have been applied [2,4,8,13].

The GFR cannot be measured easily in clinical practice, instead it is estimated (eGFR) from equations, using variables such as serum creatinine level, age, body weight, race and sex. The most recent equation, the CKD-EPI equation (Chronic Kidney Disease Epidemiology Collaboration), published in 2009, is more precise and accurate than other equations such as the Cockroft-Gault, the MDRD- (Modification of Diet in renal Disease) and the re-expressed MDRD equation, especially at GFRs > 60 mL/min/1.73 m^2^[14,15]. The CKD-EPI equation usually yields higher values for eGFR than the MDRD study equation, probably because it was developed in a more diverse study population, including participants with and without CKD. Therefore it is assumed that the CKD-EPI equation leads to smaller average bias in clinical populations with a wide range of GFRs, such as the STEMI population [16]. In a recent analysis of the PLATO trial, including 18,624 patients with an acute coronary syndrome, the CKD-EPI formula exhibited the highest prognostic value and produced a clinical relevant cut-off of 60 mL/min/1.73 m^2^[17].

The Thrombolysis In Myocardial Infarction (TIMI) risk score for STEMI is a simple arithmetic score that predicts short-term mortality based on age and clinical data on admission [18]. This score was initially developed and validated in a randomized controlled trial of patients treated with fibrinolysis but proved to be useful in patients treated with primary PCI in an observational registry (*c* statistic = 0.80 for patients treated with primary PCI in the NRMI III registry (N = 15,348)) [19].

Accordingly, we evaluated differences in prevalence of renal dysfunction at admission, defined as an eGFR < 60 mL/min/1.73 m^2^, in men and women presenting with STEMI and treated with pPCI, using the CKD-EPI equation for assessment of eGFR. We assessed the prognostic impact of renal dysfunction at admission, on top of the TIMI risk score, on in-hospital mortality in men and women and finally we evaluated whether there was an interaction between female gender and renal dysfunction regarding in-hospital mortality.

As far as we are aware this is the first study that investigates gender differences in prevalence and prognostic value of renal dysfunction, assessed by the CKD-EPI equation, in a subgroup of currently PCI-treated STEMI patients included in a national STEMI-registry.

## Methods

### Study population

The Belgian STEMI registry is a prospective observational registry of Belgian STEMI patients from 72 Belgian hospitals that contains demographics, clinical characteristics at admission, practice patterns and in-hospital outcomes. The registry is an initiative from the Belgian Working Group on Acute Cardiology (BIWAC) and is supported by the Belgian Government of Social Affairs and Public Health and the Belgian College of Cardiologists. Belgium constitutes a catchment area of 11.000.000 persons. All Belgian cardiologists working in hospitals with acute care facilities were required to prospectively collect data on all admitted STEMI patients (symptoms suspicious of acute coronary syndrome, combined with ST-segment elevation or new left bundle branch block on ECG) starting from January 2007.

**Figure 1:**
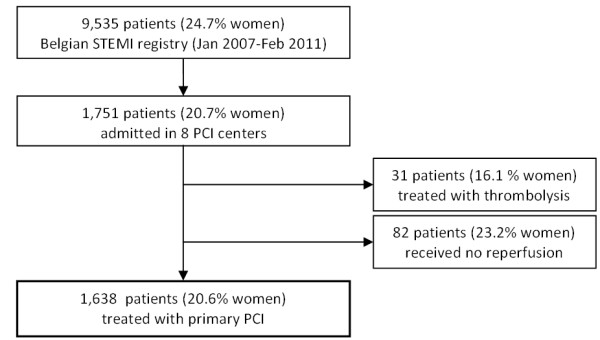
Study population, undergoing primary PCI in 8 (out of 25) selected centers.

During the period January 2007 to February 2011, a total of 9,535 (24.7% women) patients from 72 hospitals (25 with PCI facilities and 47 without PCI facilities) were prospectively included in the STEMI registry. We retrospectively collected admission values of creatinine of patients (N = 1,751; 20.7% women) admitted in the eight tertiary care centers that participated in this sub-analysis. Of these 1,638 (20.6% women) were treated with primary PCI <24 hours after hospital admission and included in the analysis (Figure 1).

A yearly audit, conducted by an external commission, of 10% of all patient files was performed to verify the validity of the data; the evaluation of these files demonstrated a 96% concordance rate between source documents and case report forms. Data were electronically collected via a protected eCRF. The database is managed by an independent electronic data-capture provider (Lambda Plus, SA, Gembloux, Belgium).

### TIMI risk score

The TIMI risk score was automatically calculated from 8 differentially weighted clinical indicators ascertained upon admission. The TIMI score ranges from 0 to 14 and is calculated as follows: age (2 points: 65–74, 3 points: 75 and older); history of angina, diabetes or hypertension (1 point); admission systolic blood pressure (BP) <100 mmHg (3 points); admission heart rate (HR) >100 beats/min (bpm) (2 points); admission Killip class > I (2 points); admission weight <67 kg (1 point); anterior infarction or left bundle branch block LBBB (1 point); and time to reperfusion therapy > 4 hours (1 point) [18].

### GFR measurement

We defined renal dysfunction as an eGFR < 60 mL/min/1.73 m^2^, corresponding to National Kidney Foundation Kidney Disease Outcomes Quality Initiative (NKF KDOQI) stages 3 to 5 of chronic kidney disease. The eGFR was calculated using the CKD-EPI equation based on the admission value of serum creatinine [14] as follows:

eGFR = 141 x min (Scr/k, 1)^a^ x max (Scr/k, 1)^-1.209^ x 0.993^age^ [x 1.018 if female] [x 1.159 if black], where Scr is serum creatinine, k is 0.7 for females and 0.9 for males, a is -0.329 for females and -0.411 for males. The eGFR could not be calculated in 11.9% of the cases, due to missing values. We found no differences in proportions of women or baseline characteristics between patients with and without missing values, except for the incidence of Cardio Pulmonary Resuscitation (CPR) (19.5% vs. 10.1%, p < 0.001) and in-hospital mortality (9.7 vs. 5.1%, p = 0.009).

### Outcome data

The primary endpoint existed of in-hospital mortality.

### Statistical analysis

We compared the baseline characteristics, including renal dysfunction at admission, and in-hospital outcomes of women with those of men. Distributions of categorical variables were compared by using the Fisher exact test. Continuous variables were evaluated for normality. The Student's *t* test was used for continuous variables with a normal distribution (presented as the mean ± SD), and the Mann Whitney-*U* test was used for continuous variables (presented as the median and interquartile range (IQR)) without a normal distribution. Multivariable logistic-regression analysis was used to determine the independent predictors of renal dysfunction at admission including following covariates in the model: gender, age, bodyweight <67 kg, history of coronary artery disease (CAD), history of peripheral artery disease (PAD), hypertension and diabetes. Separate logistic regression analyses were performed regarding in-hospital mortality for each gender, including TIMI risk score as a continuous and renal dysfunction as a dichotomous variable in the model. Multivariable logistic regression analysis was also used to assess a possible interaction between gender and renal dysfunction regarding in-hospital mortality adding renal dysfunction and gender as dichotomous variables and the product of gender and eGFR < 60 mL/min/1.73 m^2^ as an interaction term to the previous model. All multivariable analyses were based on complete patient records.

Adjusted Odds Ratios (OR) with 95% confidence intervals (CI) are reported. Statistical significance was defined as p < 0.05 or 95% confidence intervals (CI) for OR that did not include 1.0. All statistical analyses were performed using the SPSS 19 statistical software.

### Ethics approval

This study was approved by the central ethical committee of the Ghent University Hospital (2011/455). Informed consent was obtained from all patients or their legal representatives.

## Results

### Baseline characteristics

During the period between January 2007 and February 2011, 1,638 patients were treated with primary PCI in eight participating centers. Of them 338 (20.6%) were female and 1,300 (79,4%) were male. Baseline patient characteristics are shown in Table 1. As compared to men, women were on average 7 years older and more had diabetes, hypertension, and a body weight <67 kg. Furthermore, less women had previous CAD as compared to men. The history of PAD between women and men was not different. Women had longer total ischemic times. The need for CPR was not different between women and men. The Killip class and TIMI risk score at admission were higher in women vs. men. Door to balloon times (DTB) were comparable among women and men.

**Table 1:** Baseline patient characteristics (N = 1,638)

**Characteristic**	**Women (N = 338, 20.6%)**	**Men (N = 1300, 79.4%)**	**P-value**
Age, mean (SD), years	68.8 (12.7)	61.6 (12.0)	<0.001
Diabetes	21.3	13.8	<0.001
Hypertension	54.7	41.4	<0.001
Weight < 67 kg	42.3	9.8	<0.001
Previous CAD ^(a)^	16.9	22.4	0.03
Previous PAD ^(b)^	8.6	8.4	0.91
Ischemic time > 4 h ^(c)^	47	35	<0.001
Door to balloon > 120 min.	14.2	11.8	0.59
CPR ^(d)^	11.2	11.2	1.00
HR ^(e)^ > 100 bpm ^(f)^	17.2	11.5	<0.001
BP ^(g)^ < 100 mmHg	23.7	15.3	<0.001
Anterior AMI ^(h)^	49.4	45.1	0.16
Killip > I	26.6	14.3	<0.001
TIMI score, median [IQR]	5 [3.0 – 7.3]	3 [2.0 – 5.0]	<0.001
Creatinine (mg/dl) median [IQR]	0.90 [0.75-1.10]	1.00 [0.84-1.17]	<0.001
eGFR ^(i)^ <60 mL/min/1.73 m^2^	42.3	25.3	<0.001

^(a) ^coronary artery disease, ^(b) ^peripheral artery disease, ^(c) ^ischemic time = symptom to balloon time, ^(d)^ cardiopulmonary resuscitation, ^(e) ^heart rate, ^(f) ^beats per minute, ^(g) ^systolic blood pressure, ^(h) ^acute myocardial infarction, ^(i) ^estimated glomerular filtration rate.

Data are presented as percentage of patients unless otherwise specified.

### Gender and renal dysfunction

**Figure 2:**
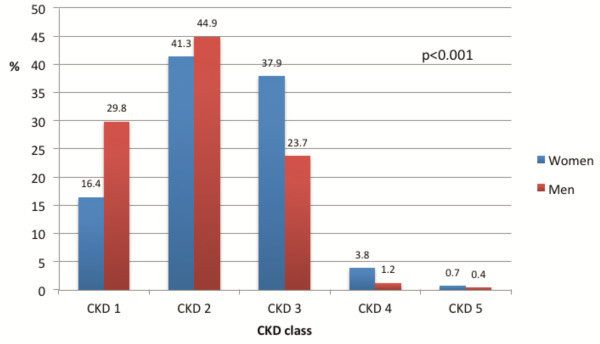
**Chronic Kidney Disease (CKD) stage at admission according to gender. CKD 1**: Chronic Kidney disease stage 1: eGFR ≥ 90 mL/min/1.73 m^2^. **CKD 2**: Chronic Kidney disease stage 2: eGFR ≥ 60 - < 90 mL/min/1.73 m^2^. **CKD 3**: Chronic Kidney disease stage 3: eGFR ≥ 30 - < 60 mL/min/1.73 m^2^. **CKD 4**: Chronic Kidney disease stage 4: eGFR ≥ 15 - < 30 mL/min/1.73 m^2^. **CKD 5**: Chronic Kidney disease stage 5: eGFR < 15 mL/min/1.73 m^2^. Blue bars = women. Red bars = men.

Median creatinine values were higher in men vs. women (median creatinine in men 1.0 [0.84-1.17] vs. 0.9 [0.75-1.10] in women, p < 0.001). Renal dysfunction, defined as an eGFR below 60 mL/min per 1.73 m^2^ or a Chronic Kidney Disease (CKD) stage 3, 4 or 5 was more frequently observed in female patients (42.3% vs. 25.3%, p < 0.001) (Figure 2). In a multivariable regression analysis, including age (/year), hypertension, diabetes, CAD or PAD and a body weight below 67 kg, female gender remained independently associated with renal dysfunction (OR 1.65, 95%CI 1.20 to 2.25, p = 0.002). Age and PAD were two other powerful determinants of renal impairment (Age: OR 1.07, 95%CI 1.05 to 1.08, p < 0.001, PAD: OR 1.89, 95% CI 1.26 to 2.84, p = 0.002). Coronary artery disease was borderline significant as a determinant of renal dysfunction (OR 1.35, 95% CI 1.00 to 1.82, p = 0.05) (Table 2).

**Table 2:** Determinants of admission renal dysfunction and Odds Ratios after multivariable adjustment

***Variable***	**OR**	**95%CI**	**p-value**
**Female gender**	1.65	1.20-2.25	0.002
**Age (/year)**	1.07	1.05-1.08	<0.001
**Weight < 67 kg**	0.87	0.61-1.23	0.87
**CAD **^**(a)**^	1.35	1.00-1.82	0.05
**PAD **^**(b)**^	1.89	1.26-2.84	0.002
**AHT **^**(c)**^	1.10	0.84-1.43	0.49
**DM **^**(dcp**^	0.97	0.69-1.36	0.87

^(a) ^coronary artery disease, ^(b) ^peripheral artery disease, ^(c) ^arterial hypertension, ^(d) ^diabetes.

### Gender, baseline renal dysfunction and in-hospital mortality

**Figure 3:**
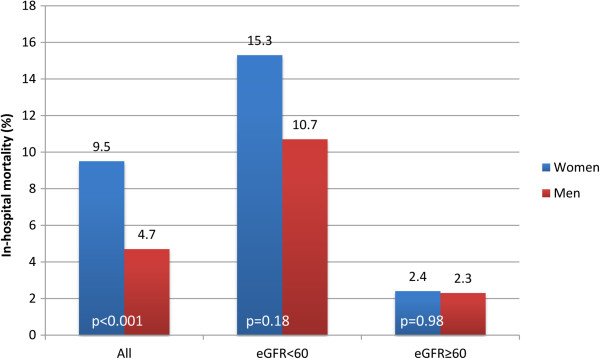
**In-hospital mortality according to gender and renal function. **eGFR = estimated glomerular filtration rate. Blue bars = women. Red bars = men.

The in-hospital mortality rate for the entire cohort of pPCI treated patients was 5.7%. Mortality in women was doubled as compared to men (9.5 vs. 4.7%), OR = 2.12 (95% CI 1.36 to 3.32, p < 0.001). Mortality in patients with renal dysfunction at the time of hospital admission was much higher compared to those without (12.0% vs. 2.3%, p < 0.001). In-hospital mortality was comparable in men and women without renal impairment: 2.3% vs. 2.4%, while there was a non-significant trend for higher mortality in women with renal dysfunction compared to men with renal dysfunction (15.3% vs. 10.7%, p = 0.18) (Figure 3).

### TIMI risk score and additive prognostic performance of baseline renal dysfunction in men and women

In a multivariable regression analysis including gender, TIMI risk score and renal dysfunction, both TIMI risk score (OR = 1.47, 95% CI 1.35-1.60) and renal dysfunction (OR 2.71, 95% CI 1.56-4.69) were strong predictors of in-hospital mortality; there was a trend towards higher mortality in women (OR 1.45, 95% CI 0.8-2.63).

Adding renal dysfunction to the TIMI risk score in men and women separately in a multivariable logistic regression model demonstrated that renal dysfunction was an independent predictor of in-hospital mortality in both men (OR (95% CI) = 2.39 (1.27-4.51), p = 0.007) and women (OR (95% CI) = 4.03 (1.26-12.92), p = 0.02), the interaction test demonstrated that this impact was comparable for men and women (p = 0.69).

## Discussion

In a sub-analysis of the Belgian STEMI registry, including 20,3% of Belgian STEMI patients undergoing pPCI for STEMI, we found that renal dysfunction at the time of hospital admission (eGFR < 60 mL/min per 1.73 m^2^ or CKD class 3 or higher), assessed by the CKD-EPI formula, was a common finding and that more women (42.3%) than men (25.3%) suffered from this condition. As expected, a CKD class 3 or higher on admission was associated with in-hospital mortality, and this independently of the TIMI risk score. Although there was a trend towards higher mortality for women with renal dysfunction compared to men with this condition, we could not demonstrate a gender difference in the impact of renal dysfunction on in-hospital mortality.

Despite the fact that male STEMI patients had a higher serum creatinine concentration at admission, the prevalence of renal dysfunction defined by an eGFR <60 mL/min per 1.73 m^2^ and assessed with the CKD-EPI formula was almost doubled in women as compared to men, even after correction for observed differences in age and risk profile between men and women. The higher serum creatinine concentrations in men can easily be explained by the larger muscle mass, resulting in greater creatinine generation, and a higher serum creatinine concentration for a given GFR. This illustrates that serum creatinine values should not be used to evaluate gender differences in the impact of renal function on outcome. It is not completely clear why the incidence of renal dysfunction is higher in women, we cannot exclude that there are unknown confounders that may explain this difference.

Many authors have demonstrated that women with STEMI have a higher risk of in-hospital mortality and some, but not all, could explain this higher mortality based on age and the presence of more comorbidities, especially hypertension and diabetes [1-5]. Lawesson et al. recently demonstrated in a small single center study, including 274 STEMI patients undergoing pPCI, that female gender was a strong and independent predictor of renal dysfunction and that renal dysfunction had a possibly higher impact on 1-year mortality in women (p for interaction = 0.08) [13]. We confirmed that renal dysfunction was more prevalent in pPCI treated women but we did not find a gender-impact on in-hospital mortality. Given the fact that the prevalence of renal dysfunction was independently related to female gender and that there was no difference in mortality between women and men with preserved renal function we speculate that renal dysfunction could be an important reason why women with STEMI die more than men.

The presented data do not reveal why renal dysfunction was associated with worse outcome. There are numerous data that demonstrate that chronic kidney disease serves as an important modifier for outcome. Renal dysfunction may serve as a surrogate marker for general health and for unknown risk factors that may explain the worse outcome. Also renal dysfunction may be associated with complications, of which development of (contrast induced) acute kidney injury (AKI) and bleeding are the most likely to occur [20]. By implementing routine calculation of eGFR, based on the CKD-EPI formula, high risk patients can be identified, and strategies for prevention of contrast induced AKI (bicarbonate administration, optimization of hemodynamic status and discontinuation of nephrotoxic drugs) [21] and other complications (e.g. bleeding) can be initiated. More over, evidence based therapies such as β-blockers, ACE-inhibitors and statins should not be withheld in this subgroup [22].

Today it is not clear whether the prognostic performance of the currently most used simple and bedside risk scores could improve by adding eGFR to the model. Kidney function, represented by various cut-offs for serum creatinine but not by eGFR, is already incorporated in the GRACE risk score [23]. Kidney function is not incorporated at all in the TIMI risk score for STEMI and this might be one of the reasons why the GRACE risk score performed better in STEMI patients in a recent meta-analysis of 15 derivation studies and 17 validation studies [24]. Given our finding that serum creatinine underestimates renal dysfunction, especially in women, future risk stratification should preferably use the eGFR based kidney function estimates such as the CKD-EPI equation.

### Strenghts and limitations

This study is unique as it represents the first dataset that links gender, renal dysfunction, assessed by the CKD-EPI equation, and outcomes in a subgroup of PCI-treated STEMI patients included in the Belgian STEMI registry. As 71.2% of this Belgian STEMI cohort presented with an eGFR > 60 mL/min/1.73 m^2^, the use of the CKD-EPI equation was appropriate.

The study has some limitations. First, we only studied patients who underwent pPCI in a subgroup of 8 tertiary care centers that participated in the Belgian STEMI registry; since it has been demonstrated that patients with renal failure have less access to invasive therapy, a selection bias is not excluded [25]. Second, it is uncertain whether renal dysfunction at the time of hospital admission represents a steady state condition of chronic kidney disease or whether there was also a component of AKI. Third, we only collected data on in-hospital mortality rates; hence, it remains unclear whether our results can be extrapolated to long-term outcomes. Finally, it is not clear how missing values could have influenced our results, however we found no differences in proportions of women and baseline characteristics, except for a higher incidence of CPR in the group with missing values. This and the higher in-hospital mortality in this group suggest that these patients were more severely ill and probably also had renal dysfunction at the time of hospital admission, which would have reinforced our findings.

## Conclusions

Renal dysfunction, defined as an eGFR < 60 mL/kg per 1.73 m^2^ was common in a subgroup of patients undergoing primary PCI for STEMI and female gender was independently associated with this condition. Renal dysfunction, independent of the TIMI risk score, was associated with higher in-hospital mortality rates in both men and women. There was no gender difference in the prognostic impact of renal dysfunction regarding in-hospital mortality. Glomerular filtration rates should routinely be assessed at baseline in these patients and these and not serum creatinine values should be accounted for when evaluating gender differences in outcome after STEMI. Whether eGFR, assessed by the CKD-EPI equation, could improve the prognostic performance of currently used bedside risk stratification models remains to be elucidated.

## Competing interests

The authors declare that there are no disclosures related to the content of this article.

## Authors’ contributions

SG designed the study. Data were collected by all authors but DDB. Data-analysis was performed by SG and DDB. The article was written by SG, MC and DDB and critically reviewed by all other authors. All authors read and approved the final manuscript.

## Pre-publication history

The pre-publication history for this paper can be accessed here:

http://www.biomedcentral.com/1471-2369/14/62/prepub
